# Preclinical characterization of [^18^F]T-008, a novel PET imaging radioligand for cholesterol 24-hydroxylase

**DOI:** 10.1007/s00259-021-05565-z

**Published:** 2021-10-15

**Authors:** Tatsuki Koike, Cristian C. Constantinescu, Shuhei Ikeda, Toshiya Nishi, Eiji Sunahara, Maki Miyamoto, Patricia Cole, Olivier Barret, David Alagille, Caroline Papin, Thomas Morley, Krista Fowles, John Seibyl, Gilles Tamagnan, Takanobu Kuroita

**Affiliations:** 1 Takeda Pharmaceutical Company Limited, 26-1 Muraoka-Higashi, 2-Chome, Fujisawa, Kanagawa 251-8555 Japan; 2 Invicro, LLC, New Haven, CT USA; 3 Takeda Pharmaceuticals International Co, Cambridge, MA USA; 4 Yale PET Center, New Haven, CT USA

**Keywords:** [^18^F], PET, Cholesterol 24-hydroxylase, CH24H, Soticlestat

## Abstract

**Purpose:**

Cholesterol 24-hydroxylase (CH24H) is a brain-specific enzyme that plays a major role in brain cholesterol homeostasis by converting cholesterol into 24*S*-hydroxycholesterol. The selective CH24H inhibitor soticlestat (TAK-935) is being pursued as a drug for treatment of seizures in developmental and epileptic encephalopathies. Herein, we describe the successful discovery and the preclinical validation of the novel radiolabeled CH24H ligand (3-[^18^F]fluoroazetidin-1-yl){1-[4-(4-fluorophenyl)pyrimidin-5-yl]piperidin-4-yl}methanone ([^18^F]T-008) and its tritiated analog, [^3^H]T-008.

**Methods:**

In vitro autoradiography (ARG) studies in the CH24H wild-type (WT) and knockout (KO) mouse brain sections were conducted using [^3^H]T-008. PET imaging was conducted in two adult rhesus macaques using [^18^F]T-008. Each macaque received two test–retest baseline scans and a series of two blocking doses of soticlestat administered prior to [^18^F]T-008 to determine the CH24H enzyme occupancy. PET data were analyzed with Logan graphical analysis using plasma input. A Lassen plot was applied to estimate CH24H enzyme occupancy by soticlestat.

**Results:**

In ARG studies, binding of [^3^H]T-008 was specific to CH24H in the mouse brain sections, which was not observed in CH24H KO or in wild-type mice after pretreatment with soticlestat. In rhesus PET studies, the rank order of [^18^F]T-008 uptake was striatum > cortical regions > cerebellum, which was consistent with CH24H distribution in the brain. Pre-blocking with soticlestat reduced the maximum uptake and increased the washout in all brain regions in a dose-dependent manner. Calculated global occupancy values for soticlestat at a dose of 0.89 mg/kg were 97–98%, indicating maximum occupancy.

**Conclusion:**

The preclinical in vitro and in vivo evaluation of labeled T-008 demonstrates that [^18^F]T-008 is suitable for imaging CH24H in the brain and warrants further studies in humans.

**Graphical abstract:**

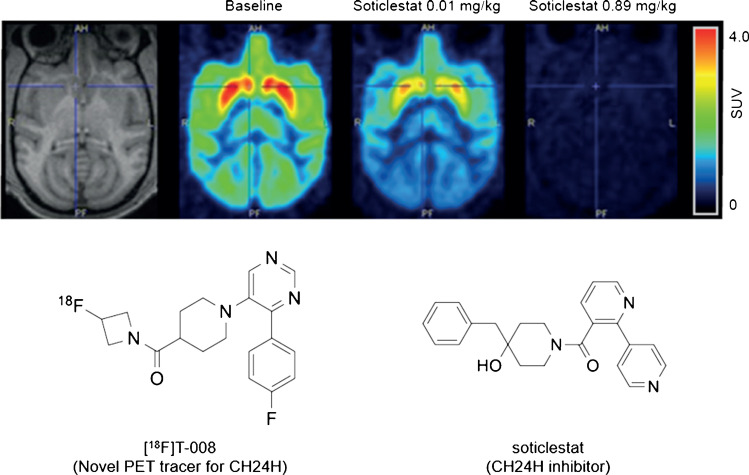

**Supplementary Information:**

The online version contains supplementary material available at 10.1007/s00259-021-05565-z.

## Introduction

Cholesterol homeostasis is a highly regulated process and plays fundamental roles in maintaining a variety of physiological functions in the brain. Cholesterol 24-hydroxylase (CH24H) is a brain-specific cytochrome P450 family enzyme encoded by *CYP46A1* which converts cholesterol to 24*S*-hydroxycholesterol, and is the dominant of cholesterol catabolic enzyme in the brain [1]. It has been reported that a *CH24H* gene polymorphism is associated with the risk of Alzheimer’s disease (AD) [2, 3]. In patients with AD, 24*S*-hydroxycholesterol levels in cerebrospinal fluid were found to be elevated compared with in healthy control subjects [4–6]. Furthermore, 24*S*-hydroxycholesterol is reported to modulate various biological functions, including facilitation of *N*-methyl-d-aspartate (NMDA) signaling, inflammation, oxidative stress, and necroptosis [7–12]. This growing body of evidence has attracted considerable attention for the development of a modulator of CH24H as a therapeutic drug target for the central nervous system as well as a basic research tool to investigate the function of CH24H in the brain. Recently, we have reported that soticlestat ((4-benzyl-4-hydroxypiperidin-1-yl)(2,4′-bipyridin-3-yl) methanone, also known as TAK-935; Supplemental Fig. 1) is a potent and selective inhibitor for CH24H [12]. Preclinical studies in mice suggested that soticlestat-mediated inhibition of CH24H may have therapeutic potential for diseases associated with neural hyperexcitation. Soticlestat is currently being investigated as a drug for treatment of Dravet syndrome and Lennox-Gastaut syndrome with a novel mechanism of action [13–15].

Positron emission tomography (PET) has long been used as a noninvasive molecular imaging modality that allows quantification of physiological and pathological processes in living subjects. PET imaging using a specific radiotracer for CH24H can potentially assess the expression levels of CH24H and their alterations in homeostatic and disease conditions. In addition, this modality can be applied to determine occupancy of CH24H by therapeutic CH24H inhibitors such as soticlestat, competing with PET imaging agents for a binding pocket; this application would be of great use for assessing target engagement of CH24H-targeting drugs. Recently, ^11^C-labeled inhibitor targeted for CH24H was reported [16]. However, PET imaging studies in mice revealed that the ^11^C-labeled compound exhibited low uptake in the brain and marginal binding specificity in vivo.

**Fig. 1:**
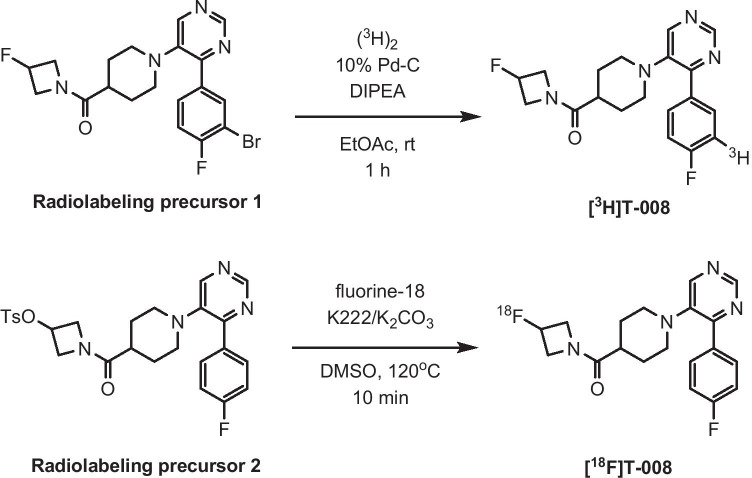
Radiosynthesis of [^3^H]T-008 and [^18^F]T-008. DMSO, dimethyl sulfoxide; DIPEA, N,N-diisopropylethylamine; EtOAc, ethyl acetate; K222, Kryptofix 222; rt, room temperature; Ts, *p*-toluenesulfonyl

T-008, (3-fluoroazetidin-1-yl){1-[4-(4-fluorophenyl)pyrimidin-5-yl]piperidin-4-yl}methanone (Supplemental Fig. 1), is a specific inhibitor for CH24H discovered as a result of iterative medicinal chemistry efforts. In this study, we developed and characterized [^3^H]T-008 and [^18^F]T-008 (Fig. 1) as the first and specific radiotracer for CH24H. The CH24H-specificity of [^3^H]T-008 was investigated by in vitro autoradiography (ARG) studies using CH24H wild-type (WT) and knockout (KO) mouse brain sections. [^18^F]T-008 was also evaluated as a PET radiotracer for in vivo imaging of CH24H and determination of the occupancy of CH24H by soticlestat in nonhuman primates (NHP). In addition, whole-body PET scans were conducted to assess the biodistribution and dosimetry of [^18^F]T-008 for the purpose of the clinical application of this radiotracer.

## Materials and methods

### Animals

Male CH24H WT and KO mice were obtained from Takeda Rabics Limited. CH24H KO mice harboring a null allele were of mixed strain background (C57BL/6 J;129S6/SvEv) [17]. Male imprinting control region (ICR) mice were supplied by CLEA Japan, Inc. Animals were housed in a light-controlled room (12-h light/12-h dark cycle with lights on at 7:00 AM). The care and use of these animals and the experimental protocols used in this research were approved by the Experimental Animal Care and Use Committee of Takeda Pharmaceutical Company Limited. Adult rhesus macaques (*Macaca mulatta*, 6–9 kg) housed at Yale University School of Medicine were used for PET studies. All experiments were conducted in accordance with Federal regulations and with institutional approval. Animal care approval and oversight for this study were provided by the Yale University Institutional Animal Care and Use Committee (IACUC).

### Radioligands and chemicals

Soticlestat was prepared according to the procedure reported previously [12]. T-008 and the radiolabeling precursors were synthesized by Takeda Pharmaceutical Company Limited (Supplemental Fig. 2). Synthesis of [^3^H]T-008, (3-fluoroazetidin-1-yl)(1-{4-[4-fluoro(3-[^3^H])phenyl]pyrimidin-5-yl}piperidin-4-yl)methanone, was performed at Quotient Bioresearch (Radiochemicals) Limited by treatment of bromo precursor 1, {1-[4-(3-bromo-4-fluorophenyl)pyrimidin-5-yl]piperidin-4-yl}(3-fluoroazetidin-1-yl)methanone, with tritium gas using palladium on carbon as catalyst (Fig. 1). The specific radioactivity and radiochemical purity of [^3^H]T-008 were 703 GBq/mmol and 99.7%, respectively. [^18^F]T-008, (3-[^18^F]fluoroazetidin-1-yl){1-[4-(4-fluorophenyl)pyrimidin-5-yl]piperidin-4-yl}methanone was synthesized from a solution of the tosylate precursor 2, 1-((1-(4-(4-fluorophenyl)pyrimidin-5-yl)piperidin-4-yl)carbonyl)azetidin-3-yl-4-methylbenzenesulfonate, in acetonitrile heated to 120 °C for 10 min, with fluorine-18 in the presence of potassium carbonate and Kryptofix 222 in dimethysulfoxide (Fig. 1). Purification was performed by high-performance liquid chromatography (HPLC) on a Phenomenex Luna C18(2) column (10 μm, 10 × 250 mm) eluted with a mixture of acetonitrile and water (35/65, v/v) at a flow rate of 4 mL/min. The product fraction was diluted with water for injection and loaded onto a solid-phase extraction (SPE; Sep-Pak® tC18 Light) cartridge. The radiolabeled product was eluted from the SPE cartridge with US Pharmacopeia grade ethanol, diluted with and passed through a sterilizing 0.2-μm membrane filter into a sterile, filter-vented vial.

Quality control testing included visual inspection of appearance. Identity, chemical, and radiochemical purity were determined by HPLC; strength was measured by gamma assay; filter integrity, pyrogen content, and sterility were determined by compendial tests per USP; residual solvents were determined by gas chromatography; and pH was measured using a pH meter or pH paper. All tests, except inoculation of product in two media for sterility, were performed before the product was released. Inoculation was performed within 30 h after end of synthesis. The molar activity and radiochemical purity of [^18^F]T-008 at the end of synthesis were 179 ± 101 GBq/µmol (*n* = 8) and 99.6 ± 0.3% (*n* = 8), respectively.

### In vitro [^3^H]T-008 ARG in CH24H WT and KO mouse brain sections

Sagittal frozen sections of CH24H WT and KO mouse brains (three mice in each genotype) on glass slides were warmed to room temperature. The sections were preincubated twice for 5 min at room temperature in preincubation buffer (50 mM Tris–HCl pH 7.5, 1.7 mM EDTA, 6 mM MgCl_2_, 120 mM NaCl, and 0.1% BSA). The sections were pretreated with or without 10 μM soticlestat in binding buffer (preincubation buffer containing 0.03% Triton X-100) for 4 h at room temperature. The sections were then treated with 100 nM [^3^H]T-008 in binding buffer for 2 h at room temperature. The sections were rinsed twice for 10 min at 4 °C in preincubation buffer and then rapidly rinsed in ice-cold distilled water. The sections were dried overnight at room temperature and were exposed to imaging plates BAS IP TR 2040E (GE Healthcare UK Ltd) for 7 days. The imaging plates were analyzed using an image analyzer (FLA-7000, Fujifilm), and image analyzing software (ImageQuant TL, GE Healthcare UK Ltd). In an autoradiographic study of CH24H WT and KO mice, regions of interest (ROI) were placed at the whole brain in each section. [^3^H]T-008 binding in the whole brain of each group was presented as percent of total binding in the whole brain of WT mouse sections.

### Brain [^18^F]T-008 PET studies

PET scans (*n* = 8) of the brain were performed on a Siemens Focus 220 microPET camera (Siemens Healthcare Molecular Imaging) in two adult female rhesus macaques (8.9 ± 0.2 kg and 6.3 ± 0.3 kg) housed at Yale University School of Medicine; four baseline studies (two test–retest studies) and four pre-blocking studies with soticlestat at two different doses, low and high dose 0.01 and 0.89 mg/kg, respectively. Animals were first anesthetized with intramuscular ketamine (10 mg/kg) and given glycopyrrolate (0.1–0.2 mg/kg) to reduce secretions, transferred to the camera, and intubated with an endotracheal tube for continuous anesthesia with ~ 2.5% isoflurane mixed with oxygen. An intravenous catheter was used for administration of fluids for hydration. Body temperature was maintained by a heated water blanket and monitored with a rectal thermometer. Vital signs including heart rate, respiration rate, blood pressure, oxygen saturation, and body temperature were monitored continuously and recorded every 2 to 15 min during the study.

Radiotracer ( [^18^F]T-008) was injected 2 h after administration of anesthetics to allow for stabilization of the animals’ physiology. PET images were acquired over 3 h after administration of a 3-min iv bolus of 184.3 ± 5.7 MBq of [^18^F]T-008. A transmission scan with an external Germanium-68 source was performed prior to the emission scans. Images were reconstructed using filtered back-projection with standard corrections for random, scatter, and attenuation.

After radiotracer administration, radial artery blood samples were collected over 3 h. Radioactivity in whole blood and plasma was measured in all samples, and radio-metabolites were measured in a subset. Plasma protein binding free fraction (*f*_*p*_) was measured by ultrafiltration. The arterial plasma input function (AIF) corrected for radio-metabolites was used for the PET data analysis.

PET images were analyzed using PMOD software (PMOD Technologies). PET images from the first baseline study were averaged, aligned onto the subject’s T1-weighted (sagittal MPRAGE, TE = 3.34 ms, TR 2.5 s, TI = 1.1 s) magnetic resonance imaging (MRI), which in turn was co-registered to a rhesus MR brain template [18]. The combined transformation was applied to the whole PET series. All PET images from the same rhesus macaque acquired in subsequent studies (baseline retest and post drug) were rigidly matched to the baseline PET images and then co-registered to the template. Neuroanatomic substrate volumes of interest including the caudate, putamen, globus pallidus, ventral striatum, thalamus, hippocampus, cortical regions, and cerebellum were defined from a brain atlas associated with the rhesus MR template and applied to the PET series to extract the time activity curves (TACs). Curves were expressed in standardized uptake value (SUV) by normalizing by the injected dose and animal body weight.

TACs were analyzed with Logan graphical analysis (LGA) with the cutoff time, t* set to 40 min to derive the total volume of distribution, *V*_*T*_, in each region. The regional binding potential, *BP*_*ND*_, was calculated as *BP*_*ND*_ = *V*_*T*_*/V*_*ND*_ – 1, where *V*_*ND*_ is the nondisplaceable volume of distribution. *V*_*ND*_ was estimated from a Lassen plot at maximum occupancy achieved following the 0.89 mg/kg dose of soticlestat described by equation *V*_*T*_^Baseline^ – *V*_*T*_^Drug^ = *O*_*CC*_ × (*V*_*T*_^Baseline^ – *V*_*ND*_), where *V*_*T*_^Baseline^ and *V*_*T*_^Drug^ are the *V*_*T*_ at baseline and post soticlestat, respectively, and *Occ* is the global occupancy. The *x*-intercept, Int*'* of the plot is used to estimate the nondisplaceable distribution volume *V*_*ND*_ = –Int′/Occ.

Test–retest reproducibility for *V*_*T*_ was estimated in the two animals as 2 × (test – retest)/(test + retest).

The CH24H regional occupancy by soticlestat was determined as the percent change of *BP*_*ND*_:

$$\mathrm{Occupancy }(\mathrm{\%})=({{BP}_{ND}}^{\mathrm{baseline}}-{{BP}_{ND}}^{\mathrm{Drug}})/{{BP}_{ND}}^{\mathrm{baseline}}\times 100$$

The occupancy in the basal ganglia (putamen, caudate, ventral striatum, and globus pallidus), and separately for cortical regions (frontal, temporal, occipital, parietal lobes, anterior, and posterior cingulate), were fitted in GraphPad Prism (version 6.01, GraphPad Software) with a single-specific-binding site model, as a function of either the administered drug dose or the drug plasma concentration:

$$\mathrm{Occ }(\%)={Occ}^{\mathrm{max}} X/(X+K)$$

where Occ^max^ is the maximum occupancy, assumed to be 100%. *K* is either ED_50_ or EC_50_, the drug dose or the plasma concentration for 50% occupancy, respectively. *X* is either the drug dose or measured plasma level.

### Whole-body [^18^F]T-008 PET studies

Two adult rhesus monkeys, one male and one female, were used for whole-body positron emission tomography (PET) imaging on a Siemens Biograph mCT camera over 4–4.3 h following intravenous bolus injection of [^18^F]T-008 to determine the biodistribution and estimate radiation absorbed doses. The whole-body images were processed in PMOD to estimate the total number of disintegrations in each source organ, which were scaled to human and then used with OLINDA|EXM 1.1 software to estimate the radiation doses absorbed by body organs, and whole-body effective doses for both adult male and female models. A more detailed description of dosimetry methods is provided in Supplemental section.

## Results

### In vitro [^3^H]T-008 ARG in the CH24H WT and KO mouse brain sections

**Fig. 2:**
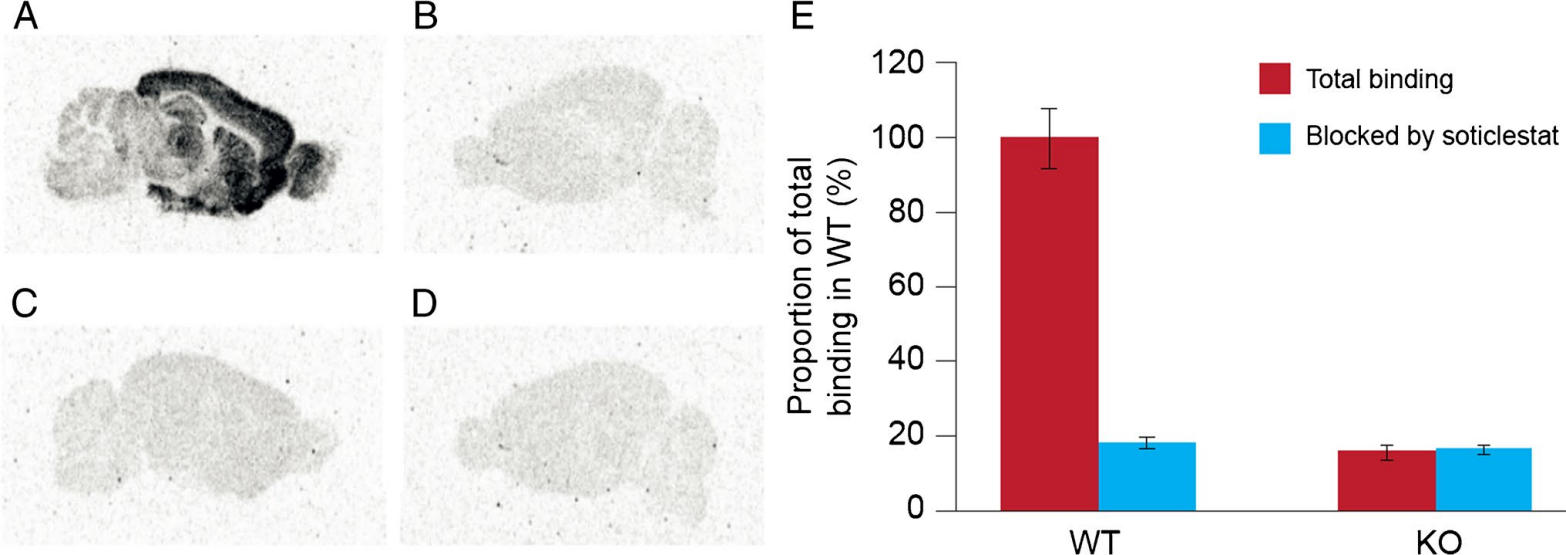
In vitro autoradiography of [^3^H]T-008 using CH24H WT and KO mouse brain sections (**A**, **B**, **C**, **D**). Total binding of [^3^H]T-008 in WT mouse brain (**A**) and KO mouse brain (**B**). Co-incubation of [^3^H]T-008 (100 nM) and soticlestat (10 μM) in WT mouse brain (**C**) and KO mouse brain (**D**). Binding ratio of [^3^H]T-008 in the whole brain. Total binding in WT mouse sections was calculated as 100% (**E**). CH24H, cholesterol 24-hydroxylase; KO, knockout; WT, wild-type

Figure 2 shows representative in vitro ARG of [^3^H]T-008 using CH24H WT and KO mouse brain sections. [^3^H]T-008 was accumulated in WT mouse brain and the distribution pattern was heterogeneous (Fig. 2A). This accumulation of [^3^H]T-008 was not observed in CH24H-KO mouse brain sections (Fig. 2B). Non-specific binding (NSB) is shown in Fig. 2C. [^3^H]T-008 binding in the whole brain of each group was calculated as percent of total binding in WT mouse sections. NSB was 18.7% ± 1.4%, and was almost equal to the binding amount of [^3^H]T-008 in CH24H-KO mouse sections in the absence (16.0% ± 1.8%, Fig. 2B) and presence (16.7% ± 1.0%, Fig. 2D) of 10 μM of soticlestat (Fig. 2E).

### Plasma [^18^F]T-008 PET analysis

Percentage of unmetabolized (intact) [^18^F]T-008 in plasma was measured by HPLC. [^18^F]T-008 was moderately metabolized (T_1/2_ ~ 15 min), with 31% ± 6% and 13% ± 5% (*n* = 8) of intact parent remaining at 30 min and 180 min post injection, respectively (Supplemental Fig. 4). Soticlestat did not change the intact tracer profile measured at baseline. Plasma-free fraction, *f*_*p*_ (fraction unbound to plasma proteins) measured by ultrafiltration was 51.4% ± 5.4% (*n* = 8).

### Brain [^18^F]T-008 PET studies

**Fig. 3:**
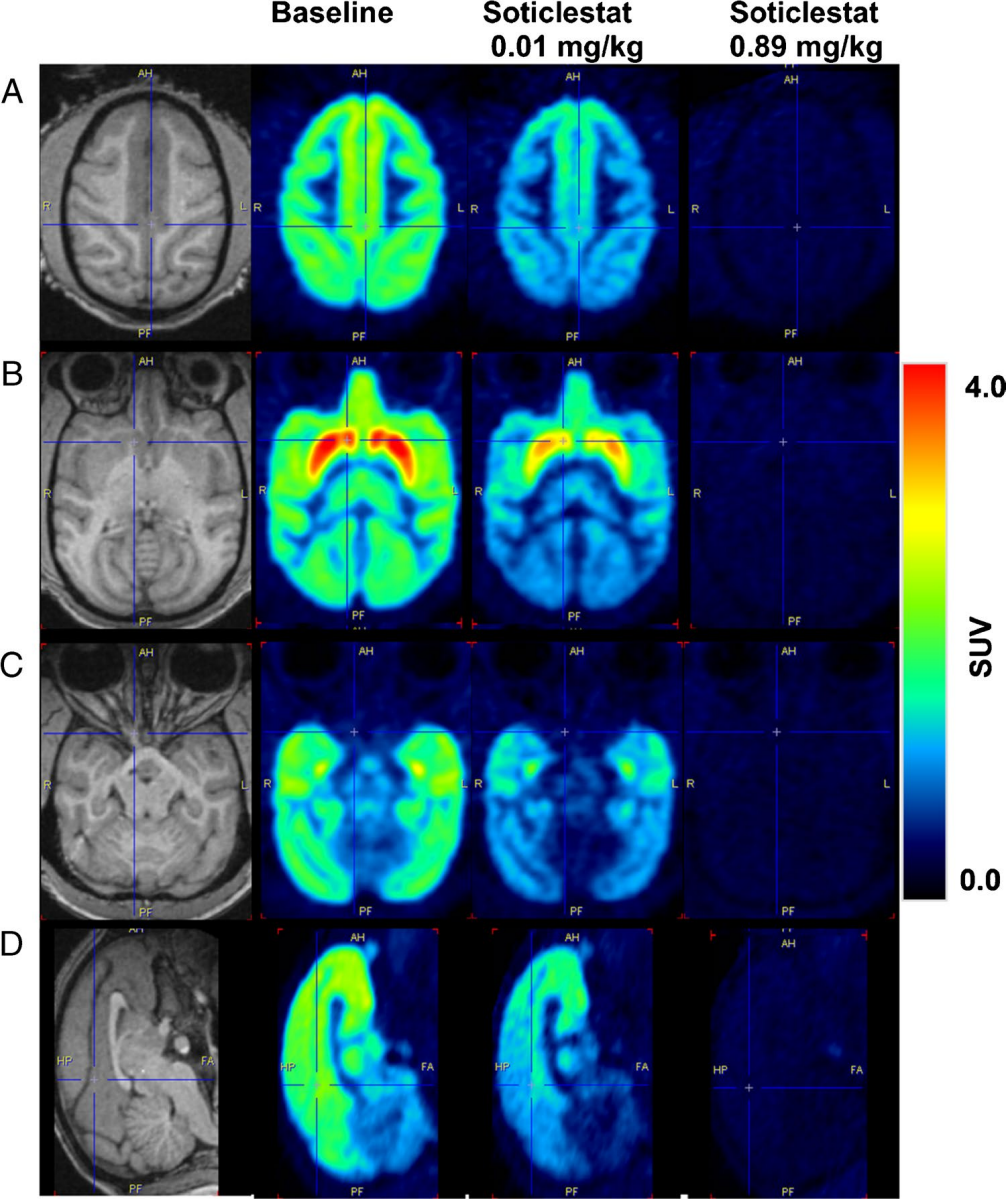
Average [^18^F]T-008 PET images normalized by the injected activity and animal weight (SUV units) over 180 min for a rhesus macaque in (**A**, **B**, **C**) axial, and (**D**) sagittal plane at baseline and post dosing with soticlestat at 0.01 mg/kg and 0.89 mg/kg. Monkey individual MRI is also shown on the left-hand side. MRI, magnetic resonance imaging; PET, positron emission tomography; SUV, standardized uptake value

**Fig. 4:**
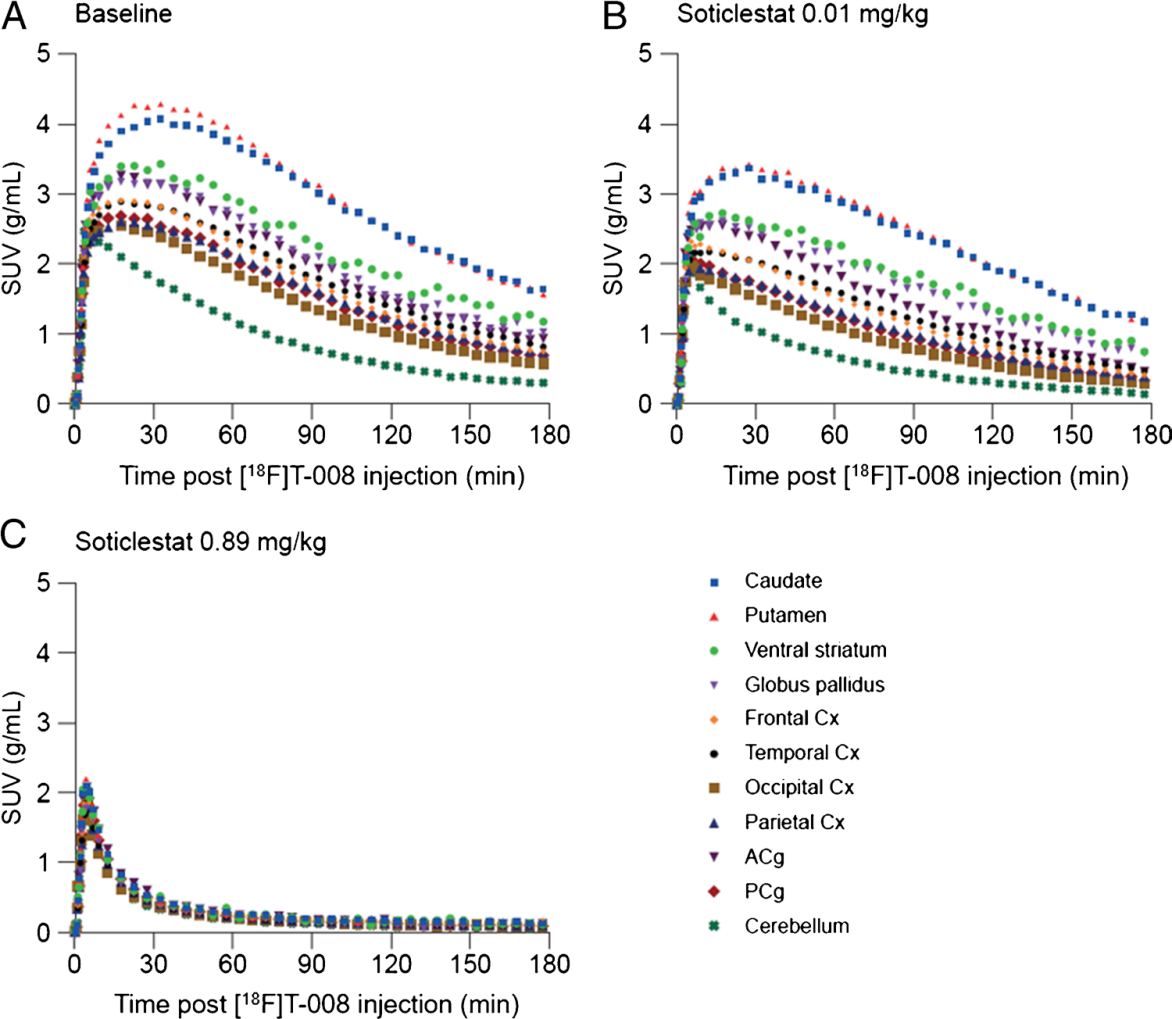
Representative time activity curves from several brain regions acquired at baseline (**A**) and during competition with 0.01 mg/kg soticlestat (**B**) and 0.89 mg/kg soticlestat (**C**). ACg, anterior cingulate gyrus; Cx, cortex; PCg, posterior cingulate gyrus; SUV, standardized uptake value

Average [^18^F]T-008 PET images showed the highest uptake in the striatal regions such as the putamen and caudate, followed by the globus pallidus, cortical regions, and other nuclei such as the thalamus (Fig. 3). The lowest uptake was seen in the cerebellum. This distribution was consistent with CH24H enzyme density obtained by western-blotting analysis (Supplemental Fig. 5). Representative TACs from several brain regions acquired at baseline and during blockade at two different soticlestat doses (0.89 and 0.01 mg/kg) are presented in Fig. 4. [^18^F]T-008 reached a peak uptake at baseline at 20–30 min after injection in the putamen, caudate, globus pallidus, and ventral striatum with a 40–45% washout at 180 min. In all other brain regions, the uptake was lower with a moderate washout, in particular in the cerebellum with a peak uptake within 5 min of injection, consistent with lower CH24H enzyme density in these regions. For the occupancy studies, pre-blocking with soticlestat reduced the maximum uptake and the SUV peak times and increased the washout in all regions in a dose-dependent fashion, with the highest dose tested of 0.89 mg/kg generating a fast washout in all regions, with very similar kinetics in all regions, indicating maximum occupancy.

Total distribution volume, *V*_*T*_, estimates (*n* = 4) ranged from 6.56 ± 0.90 in the cerebellum to 25.53 ± 5.96 in the putamen, with the rank order putamen > caudate > ventral striatum = globus pallidus >  > cortical regions > cerebellum. Test–retest reproducibility for *V*_*T*_ and *BP*_*ND*_ is summarized in Supplemental Table 1. Average test–retest variability of *V*_*T*_ estimated with LGA was 6% for rhesus 1 (R1) and 20% for rhesus 2 (R2). In the absence of an ideal reference region lacking the CH24H enzyme, *V*_*ND*_ was estimated from a Lassen plot at maximum occupancy achieved after administration of the 0.89 mg/kg dose of soticlestat (Supplemental Fig. 6). Global occupancy values for the 0.89 mg/kg dose of soticlestat were 97–98% (Table 1). *V*_*ND*_ and *V*_*T*_ in the cerebellum, a region with low [^18^F]T-008 uptake that was considered to be a potential reference region, are also listed in Table 1. The *V*_*T*_ values of the cerebellum at baseline were three to five times higher than *V*_*ND*_ values (= 1.11–2.26), which indicates that the cerebellum could not be used as reference region to calculate *BP*_*ND*_. The binding potentials at baseline and post soticlestat, and regional occupancies in caudate, putamen, ventral striatum, globus pallidus, frontal cortex, temporal cortex, occipital cortex, parietal cortex, anterior cingulate, posterior cingulate, thalamus, hippocampus, and cerebellum at the two soticlestat doses are listed in Table 2. The *BP*_*ND*_ in putamen was estimated to be 17.25 and 12.42 in the two animals.

**Table 1:** Soticlestat Global Occupancy Measured with [^18^F]T-008 and Logan graphical analysis. The nondisplaceable volumes of distribution and cerebellum at baseline and post administration of 0.89 mg/kg soticlestat are also listed

Rhesus macaques	Global occupancy (%)	*V*_*ND*_	*V*_*T*_^*Ref*^ (baseline)	*V*_*T*_^*Ref*^ (soticlestat, 0.89 mg/kg)
Rhesus 1	98	1.11	5.95	1.17
Rhesus 2	97	2.26	7.16	2.44

*V*_*ND,*_ nondisplaceable volumes of distribution; *V*_*T*_^*Ref*^, nondisplaceable volumes of cerebellum

**Table 2:** [^18^F]T-008 regional binding potentials for baseline (average test–retest) and soticlestat studies and soticlestat regional occupancies at 0.89 mg/kg and 0.01 mg/kg, respectively

Region	*BP*_*ND*_, baseline		*BP*_*ND*_, soticlestat				Occupancy			
			0.89 mg/kg	0.89 mg/kg	0.01 mg/kg	0.01 mg/kg	0.89 mg/kg	0.89 mg/kg	0.01 mg/kg	0.01 mg/kg
	R1	R2	R1	R2	R1	R2	R1	R2	R1	R2
Caudate	16.99	9.79	0.36	0.20	13.43	7.75	98%	98%	21%	21%
Putamen	17.25	12.42	0.35	0.39	13.65	9.73	98%	97%	21%	22%
Ventral striatum	12.24	8.29	0.31	0.31	9.54	6.32	97%	96%	22%	24%
Globus pallidus	11.25	10.81	0.22	0.25	8.74	8.58	98%	98%	22%	21%
Frontal cortex	8.78	4.94	0.26	0.17	5.83	3.53	97%	96%	34%	29%
Temporal cortex	9.48	5.66	0.11	0.17	6.22	4.22	99%	97%	34%	25%
Occipital cortex	7.04	3.70	0.02	0.07	3.95	2.44	100%	98%	44%	34%
Parietal cortex	8.21	4.73	0.14	0.12	4.98	3.32	98%	97%	39%	30%
Anterior cingulate	10.21	5.26	0.35	0.20	7.19	3.76	97%	96%	30%	29%
Posterior cingulate	8.07	4.50	0.21	0.17	4.72	3.02	97%	96%	41%	33%
Thalamus	7.30	3.73	0.25	-0.01	4.45	2.35	97%	100%	39%	37%
Hippocampus	9.45	4.54	0.13	0.18	6.79	3.21	99%	96%	28%	29%
Cerebellum	4.18	2.19	0.02	0.09	2.26	1.26	99%	96%	46%	43%

*BP*_*ND*_, binding potentials; *R1*, rhesus macaque 1; *R2*, rhesus macaque 2

**Fig. 5:**
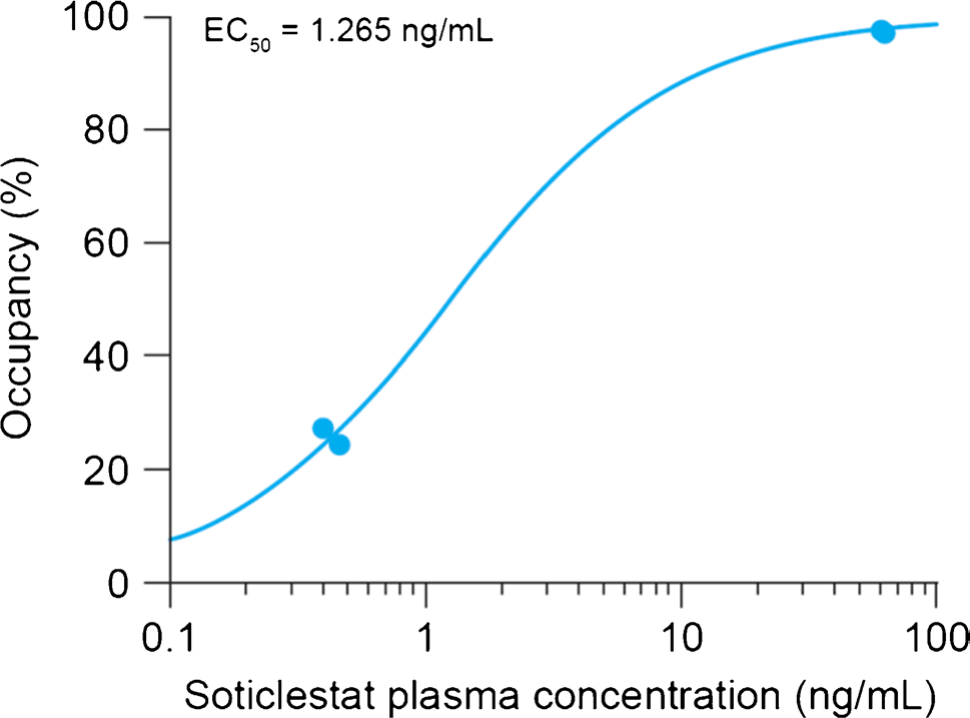
Nonlinear regression of brain CH24H occupancy as a function of soticlestat average plasma levels during imaging (30 min to 210 min post soticlestat administration). CH24H, cholesterol 24-hydroxylase; EC_50_, plasma concentration for 50% occupancy

The relationship between the average plasma concentration of soticlestat from 30 min (time of radiotracer injection) to 210-min post drug administration (*C*_*30–210*_) and brain CH24H occupancy is presented in Fig. 5. Nonlinear curve fitting revealed an EC_50_ of 1.265 ± 0.112 ng/mL.

### Dosimetry of [^18^F]T-008

[^18^F]T-008 whole-body images are shown in Supplemental Fig. 7, and the calculated absorbed doses and whole-body effective doses are presented in Supplemental Table 3. Urinary bladder received the highest radiation dose (1.49E − 01 ± 2.55E − 02). The effective dose (International Commission on Radiological Protection publication 60) was 0.0236 mSv/MBq for adult female rhesus macaques and 0.0214 mSv/MBq for adult male rhesus macaques, similar to other ^18^F-labeled radiopharmaceuticals.

## Discussion

The aim of this study was to develop a PET radiotracer for in vivo imaging of the CH24H enzyme in the brain and evaluation of central target engagement of a CH24H therapeutic agent. T-008 was selected as the most promising candidate as a result of iterative medicinal chemistry efforts. The compound was radiolabeled with tritium and evaluated with in vitro ARG using mouse brain sections. [^3^H]T-008 was accumulated in WT mouse brain and the distribution pattern was heterogeneous. The localization of [^3^H]T-008 signals agrees with immunohistochemistry of CH24H protein in mouse brain [12], supporting the binding specificity to the target. This accumulation was not observed in the presence of an excess amount of soticlestat (10 μM) as well as in ARG studies using CH24H-KO mouse brain sections, further supporting that [^3^H]T-008 accumulated in the brain slices in a CH24H-specific manner. In the saturation binding assay of [^3^H]T-008, NSB was determined in the presence of an excess amount of soticlestat and was linear over the range of concentration used. Selective and saturable bindings of [^3^H]T-008 were observed and *K*_*d*_ values was calculated as 14.0 ± 1.0 nM.

The radiosynthesis of [^18^F]T-008 was successfully achieved by nucleophilic displacement of the tosyl group in the azetidine ring of the precursor with fluorine-18. [^18^F]T-008 was synthesized with high radiochemical purity (> 99%) and high molar activity, satisfying the tracer conditions for PET imaging which require adequately low injected molar mass. [^18^F]T-008 was evaluated for the brain imaging of CH24H in NHP and showed the highest uptake in the striatal regions such as the putamen and caudate, and the lowest uptake in the cerebellum. Uptakes of [^18^F]T-008 are consistent with the CH24H enzyme density results obtained by western-blotting analysis, supporting that the uptake of [^18^F]T-008 is a CH24H-specific activity. This CH24H-specific activity could be attributed in part to the relatively lower lipophilicity of T-008 (Log D = 1.8, measured at pH 7.4) [19], which contributes to the minimum non-specific bindings of [^18^F]T-008. High expression levels of CH24H in the striatal region in NHP are also consistent with those in humans [20], suggesting that the spatial analysis of tracer uptake is of clinical relevance. Therefore, the NHP model characterized in this study is a useful translational research tool for noninvasive visualization of CH24H for various applications including enzyme expression analysis and evaluation of enzyme occupancy by a CH24H inhibitor.

The volume of distribution of [^18^F]T-008 in NHP brains was robustly estimated with LGA, which uses an AIF in the absence of an ideal tissue reference region. The test–retest reproducibility of *V*_*T*_ estimated with LGA was good, and average test–retest variability was less than 20% in two animals.

The selectivity and specificity of [^18^F]T-008 for CH24H were demonstrated against the structurally unrelated CH24H inhibitor soticlestat. Administration of soticlestat at 0.89 mg/kg produced maximum occupancy, which allowed an accurate estimation of *V*_*ND*_ from the Lassen plot. At a dose of soticlestat 0.01 mg/kg, measured CH24H occupancies were about 20% in high-binding regions but increased to up to 40–60% in lower binding regions such as cortical regions and the cerebellum. These findings were considered to be an effect of low displacement in regions with low specific binding, and, in the absence of data at intermediate doses of soticlestat, are not evidence of differential CH24H occupancy. *V*_*T*_ values of cerebellum at baseline were ~ 5 times higher than the nondisplaceable volume of distribution, *V*_*ND*_, which demonstrates that cerebellum could not be used as reference region to calculate *BP*_*ND*_. Following a thorough examination, it was found that soticlestat blocked the signal in all brain regions, which supports the absence of an appropriate reference region devoid of CH24H and indicates that quantification using AIF will be required in future studies.

In NHP occupancy studies, plasma concentration of soticlestat was measured and nonlinearly modeled against plasma concentration with a single-specific-binding site model, which assumes a direct relationship. This result suggests that [^18^F]T-008 is a potential tool to investigate the occupancy of CH24H drug candidates in clinical trials, which provide critical information on target engagement and the selection of optimal dose for efficacy.

The biodistribution studies showed that the organs with the highest radioactivity included urinary bladder, intestine, liver, brain, kidneys, and gallbladder. Radiotracer was eliminated via both renal and hepatobiliary routes. Urinary bladder received the highest radiation dose. The estimated radiation exposure (0.021–0.0248 mSv/MBq) is in line with other ^18^F-labeled agents (e.g., 0.019 mSv/MBq for fluorodeoxyglucose, ^18^F-FDG) and would allow several scans to be performed in the same subject at an injected activity of 185 MBq.

## Conclusion

In this study, we have demonstrated that [^18^F]T-008, a first PET tracer for CH24H, is suitable for imaging CH24H in the brain. In vitro ARG studies using CH24H WT and KO mouse brain sections demonstrated the specificity of [^3^H]T-008 to CH24H. The specificity of the radiotracer was also demonstrated through competition of [^18^F]T-008 with soticlestat, a selective CH24H inhibitor. Robust quantification of [^18^F]T-008 in NHP could be achieved in the absence of a tissue reference region. These observations support the use of [^18^F]T-008 (also known as [^18^F]MNI-792) for PET imaging of CH24H in healthy and diseased conditions in humans and for in vivo evaluations of interactions between CH24H and candidate therapeutics.

## Supplementary Information

Below is the link to the electronic supplementary material.

Supplementary file1 (DOC 1701 KB)
