# Supplementary material for: Preclinical characterization of [18F]T-008, a novel PET imaging radioligand for cholesterol 24-hydroxylase
Source: Eur J Nucl Med Mol Imaging. 2021 Oct 15;49(4):1148–56. doi: 10.1007/s00259-021-05565-z (PMC8921165; doi:10.1007/s00259-021-05565-z)
Supplement: Supplementary file 1 — Supplementary file1 (DOC 1701 KB) [file 259_2021_5565_MOESM1_ESM.doc]

**Supplememtal information**

Preclinical Characterization of [^18^F]T-008, a Novel PET Imaging Radioligand for Cholesterol 24-Hydroxylase

Tatsuki Koike^1^, Cristian C. Constantinescu^2^, Shuhei Ikeda^1^, Toshiya Nishi^1^, Eiji Sunahara^1^, Maki Miyamoto^1^, Patricia Cole^3^, Olivier Barret^2^, David Alagille^2^, Caroline Papin^2^, Thomas Morley^2^, Krista Fowles^4^, John Seibyl^2^, Gilles Tamagnan^2,4^, and Takanobu Kuroita^1^

*^1^Takeda Pharmaceutical Company Limited, Kanagawa, Japan,*

*^2^Invicro, LLC, New Haven, Connecticut, United States,*

*^3^Takeda Pharmaceuticals International Co, Cambridge, Massachusetts, United States,*

*^4^Yale PET Center, New Haven, Connecticut, United States*

**Supplemental** **Figure 1.** Structures of soticlestat, T-008, [^3^H]T-008, and [^18^F]T-008


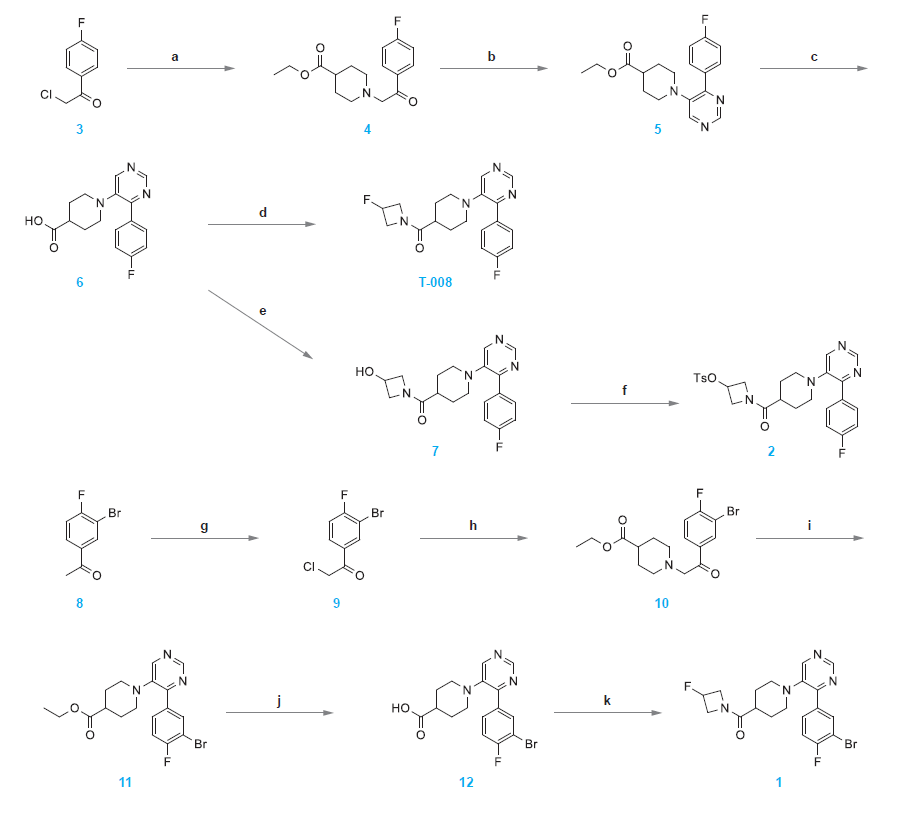


**Supplemental Figure 2**. Synthesis of T-008 and radiolabeling precursor 1 and 2^a^

^a^Reagents and conditions: (a) ethyl isonipecotate, K_2_CO_3_, MeCN, rt; (b) (1) DMF-DMA, reflux, (2) formamidine acetate, DIPEA, n-BuOH, 100^o^C; (c) 2M NaOH, THF/MeOH, rt; (d) 3-fluoroazetidine hydrochloride, HATU, DIPEA, DMF, rt; (e) azetidin-3-ol hydrochloride, HATU, DIPEA, DMF, rt; (f) TsCl, Et_3_N, trimethylamine hydrochloride, MeCN, rt; (g) NCS, *p*-TsOH-H_2_O, MeCN, reflux; (h) ethyl isonipecotate, K_2_CO_3_, MeCN, rt; (i) (1) DMF-DMA, reflux, (2) formamidine acetate, DIPEA, n-BuOH, 100^o^C; (j) 2 M NaOH, THF/EtOH, rt; (k) azetidin-3-ol hydrochloride, BuOH, butanol; DIPEA, N,N-diisopropylethylamine; DMA, dimethylacetamide; DMF, dimethylformamide; EtOH, ethanol; Et_3_N, triethylamine ; HATU, hexafluorophosphate azabenzotriazole tetramethyl uronium; MeCN, acetonitrile; MeOH, methanol; NCS, N-chlorosuccinimide; THF, tetrahydrofuran; TsCl, p-toluenesulfonyl chloride; TsOH, p-toluenesulfonic acid.


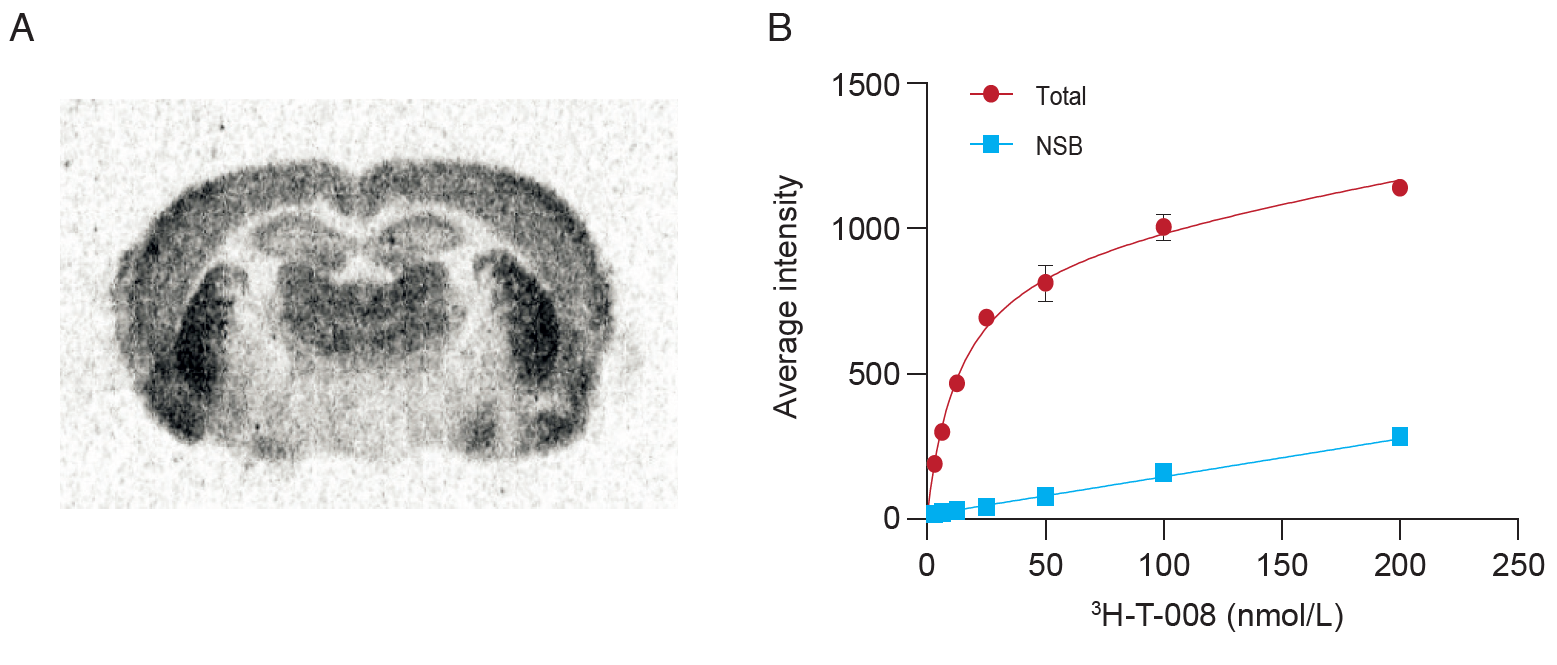


**Supplemental Figure 3.** Saturation binding of [^3^H]T-008 in mouse brain sections. Regions of interest were placed at whole area of brain in the autoradiograms (A). Specific binding of [^3^H]T-008 was calculated from the difference between total binding and NSB. NSB was determined in the presence of an excess amount
of soticlestat (10 μM). The saturation binding curves of whole brain were analyzed by nonlinear
regression. Selective and saturable bindings of [^3^H]T-008 were observed with *K*_d_ values of 14.0 ± 1.0 nM. (B). NSB, nonspecific binding.


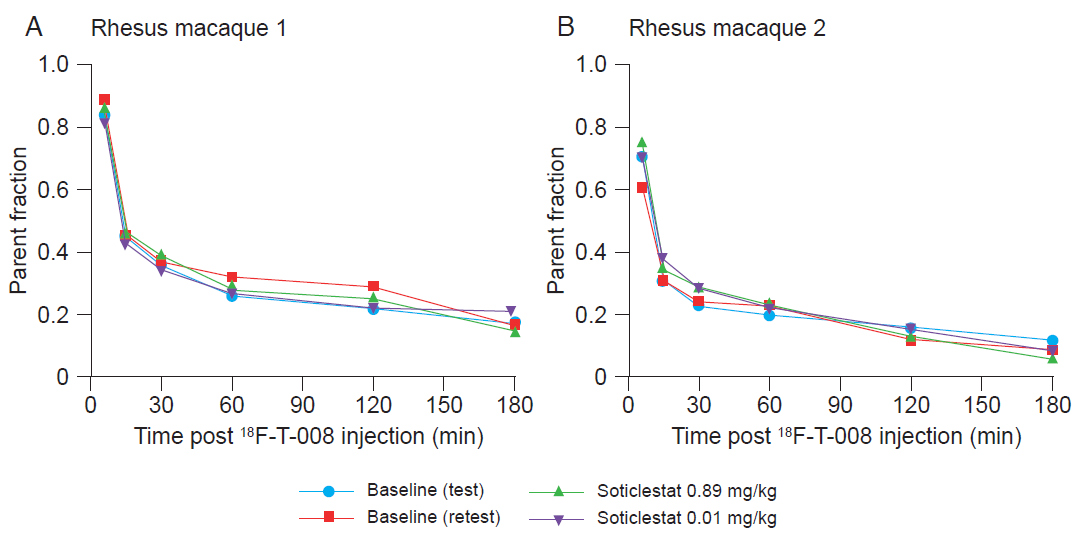


**Supplemental Figure 4**. [^18^F]T-008 plasma parent fraction measured during in vivo scans at baseline and during competition with 0.01 mg/kg and 0.89 mg/kg soticlestat in two rhesus monkeys.


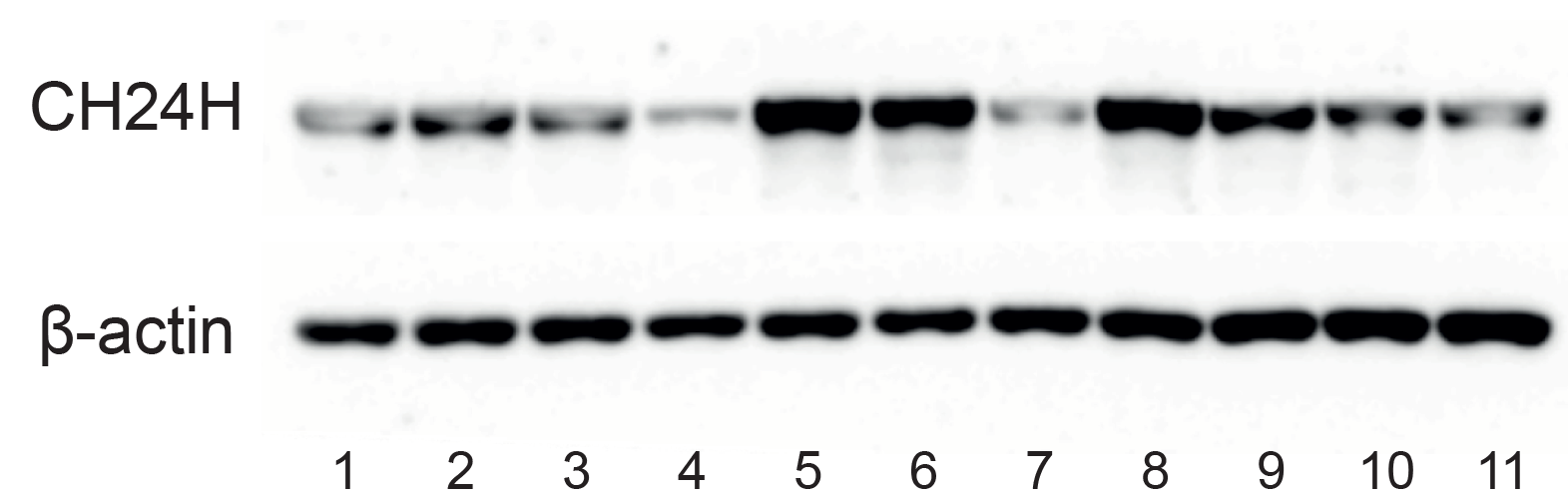


**Supplemental** **Figure 5.** Western-blotting analysis using cynomolgus monkeys. 1. posterior cingulate cortex, 2. anterior cingulate cortex, 3. frontal lobe, 4. cerebellum, 5. putamen, 6. thalamus, 7. pariental lobe, 8. caudate nucleus, 9. temporal lobe, 10. occipital lobe, 11. hippocampus. CH24H, cholesterol 24-hydroxylase.


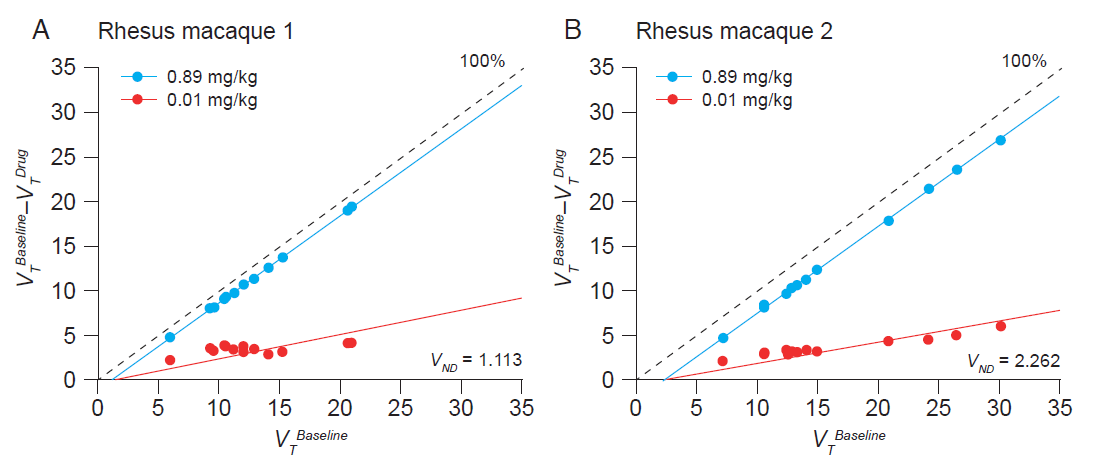


**Supplemental Figure 6**. CH24H occupancy plots from two monkeys imaged with [^18^F]T-008 at 30 min post dosing with 0.01 mg/kg and 0.89 mg/kg soticlestat. Dotted lines represent 100% occupancy and
zero *V_ND_*.


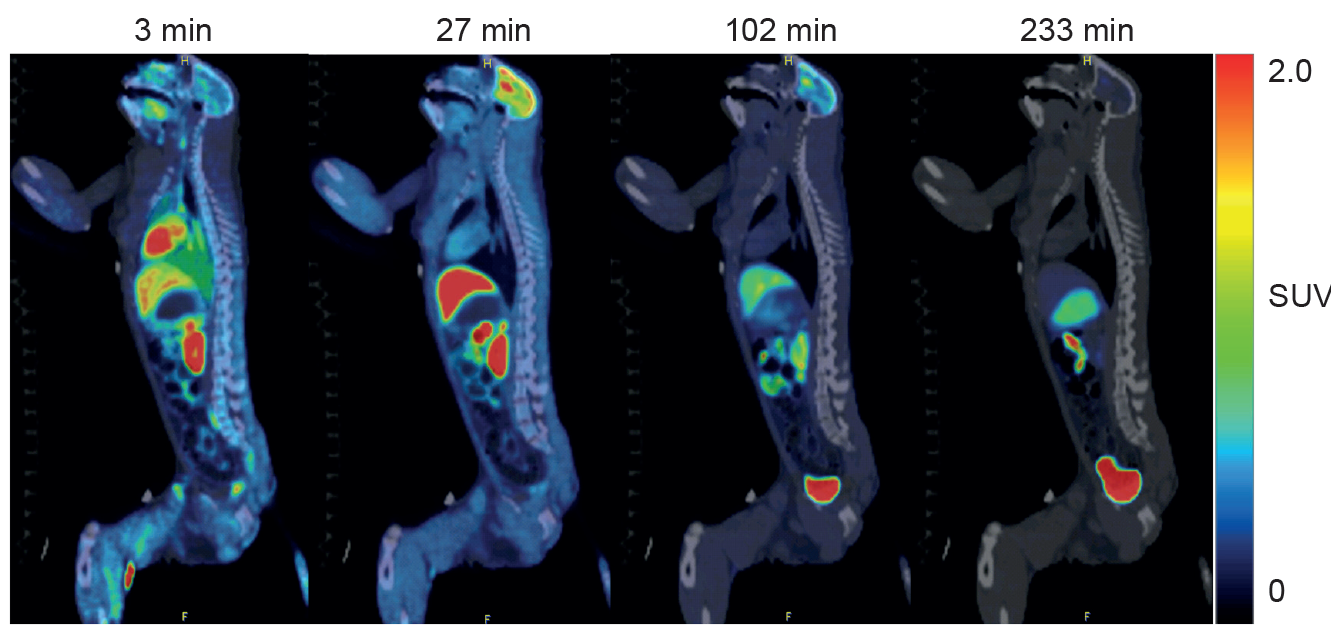


**Supplemental Figure 7.** Dynamic [^18^F]T-008 whole-body PET imaging (sagittal view) of a male rhesus macaque fused with the static whole-body CT image. Each image shows the evolution of the activity concentration over time (scans 2, 7, 12, and 17). CT, computed tomography; PET, positron emission tomography; SUV, standardized uptake value.

**Supplemental Table 1.** Test–retest reproducibility for *V_T_* (mL/cm^3^).

| **Region** | ***V_T_*, test** | | ***V_T_*, retest** | | **%TRT** | |
| --- | --- | --- | --- | --- | --- | --- |
|  | R1 | R2 | R1 | R2 | R1 | R2 |
| Caudate | 20.05 | 21.60 | 21.26 | 26.78 | −6% | −21% |
| Putamen | 20.14 | 26.78 | 21.78 | 33.41 | −8% | −22% |
| Ventral striatum | 15.03 | 18.91 | 15.38 | 22.74 | −2% | −18% |
| Globus pallidus | 13.27 | 23.54 | 14.86 | 29.41 | −11% | −22% |
| Frontal cortex | 11.08 | 12.21 | 11.38 | 14.44 | −3% | −17% |
| Temporal cortex | 11.66 | 13.23 | 12.40 | 16.64 | −6% | −23% |
| Occipital cortex | 8.89 | 9.28 | 9.56 | 11.82 | −7% | −24% |
| Parietal cortex | 10.29 | 11.55 | 10.85 | 14.17 | −5% | −20% |
| Anterior cingulate | 12.73 | 12.87 | 13.01 | 15.18 | −2% | −16% |
| Posterior cingulate | 10.09 | 10.96 | 10.74 | 13.72 | −6% | −22% |
| Thalamus | 9.26 | 9.71 | 9.81 | 11.50 | −6% | −17% |
| Hippocampus | 11.65 | 11.11 | 12.35 | 13.75 | −6% | −21% |
| Cerebellum | 5.76 | 6.49 | 6.14 | 7.84 | −6% | −21% |

*V_T_*, total volume of distribution; %TRT, percentage test–retest; R1, rhesus macaque 1; R2, rhesus macaque 2.

**Supplemental Table 2.** Scaling factors for different organs determined from their percentage contribution to total body weight for rhesus monkeys and humans.

| **Organ** | **Proportion of total  body weight (%)**  **Monkey** | **Proportion of total body weight (%)**  **Human** | **Correction factor** |
| --- | --- | --- | --- |
| Brain | 1.4 | 2 | 1.41 |
| Heart wall | 0.4 | 1.2 | 2.60 |
| Liver | 2.3 | 2.5 | 1.05 |
| Gallbladder | <0.1^*^ | <0.1 | 0.98 |
| Gastrointestinal tract | 3.3 | 1.3 | 0.43 |
| Spleen | 0.1 | 0.2 | 2.75 |
| Lungs | 0.8 | 1.6 | 1.96 |
| Kidneys | 0.4 | 0.4 | 1.09 |
| Urinary bladder^†^ | 0.1 | 0.1 | 1.00 |
| Uterus | 0.20 | 0.11 | 0.53 |
| Remaining | 84.9 | 78.2 | 0.92 |

^*^Gallbladder weight in monkeys estimated from gallbladder/(liver + gallbladder) in humans since gallbladder + liver was reported for monkeys.

^†^No urinary bladder weight available for monkeys; equal proportions were assumed.

**Supplemental Table 3.** Radiation absorbed dose estimates (in mSv/MBq) for [^18^F]T-008 in target organs of adult male and female models.

| **Target organ** | **Absorbed doses (mSv/MBq)** | | |
| --- | --- | --- | --- |
|  | **Male rhesus** | **Female rhesus** | **Mean ± SD** |
| Adrenals | 1.20E−02 | 1.65E−02 | 1.43E−02 ± 3.18E−03 |
| Brain | 1.25E−02 | 1.73E−02 | 1.49E−02 ± 3.39E−03 |
| Breasts | 7.81E−03 | 1.09E−02 | 9.36E−03 ± 2.18E−03 |
| Gallbladder wall | 1.95E−02 | 2.47E−02 | 2.21E−02 ± 3.68E−03 |
| LLI wall | 2.05E−02 | 2.57E−02 | 2.31E−02 ± 3.68E−03 |
| Small intestine | 2.92E−02 | 4.12E−02 | 3.52E−02 ± 8.49E−03 |
| Stomach wall | 1.16E−02 | 1.59E−02 | 1.38E−02 ± 3.04E−03 |
| ULI wall | 3.09E−02 | 4.42E−02 | 3.76E−02 ± 9.40E−03 |
| Heart wall | 1.71E−02 | 2.10E−02 | 1.91E−02 ± 2.76E−03 |
| Kidneys | 2.90E−02 | 2.21E−02 | 2.56E−02 ± 4.88E−03 |
| Liver | 1.95E−02 | 3.31E−02 | 2.63E−02 ± 9.62E−03 |
| Lungs | 1.10E−02 | 1.55E−02 | 1.33E−02 ± 3.18E−03 |
| Muscle | 1.05E−02 | 1.36E−02 | 1.21E−02 ± 2.19E−03 |
| Ovaries | 1.74E−02 | 2.14E−02 | 1.94E−02 ± 2.83E−03 |
| Pancreas | 1.27E−02 | 1.71E−02 | 1.49E−02 ± 3.11E−03 |
| Red marrow | 1.15E−02 | 1.39E−02 | 1.27E−02 ± 1.70E−03 |
| Osteogenic cells | 1.54E−02 | 2.15E−02 | 1.85E−02 ± 4.31E−03 |
| Skin | 7.62E−03 | 1.02E−02 | 8.91E−03 ± 1.82E−03 |
| Spleen | 1.58E−02 | 1.36E−02 | 1.47E−02 ± 1.56E−03 |
| Testes | 1.01E−02 |  | 1.01E−02 |
| Thymus | 9.86E−03 | 1.37E−02 | 1.18E−02 ± 2.72E−03 |
| Thyroid | 9.36E−03 | 1.20E−02 | 1.07E−02 ± 1.87E−03 |
| Urinary bladder wall | 1.67E−01 | 1.31E−01 | 1.49E−01 ± 2.55E−02 |
| Uterus | 2.24E−02 | 1.78E−02 | 2.01E−02 ± 3.25E−03 |
| Total body | 1.13E−02 | 1.49E−02 | 1.31E−02 ± 2.55E−03 |
| EDE (ICRP-26) | 2.57E−02 | 2.80E−02 | 2.69E−02 ± 1.63E−03 |
| ED (ICRP-60) | 2.14E−02 | 2.36E−02 | 2.25E−02 ± 1.56E−03 |

SD, standard deviation; LLI, lower large intestine; ULI, upper large intestine; ICRP-26, International Commission on Radiological Protection publication 26; ICRP-60, International Commission on Radiological Protection publication 60.

**Radiation dosimetry calculation**

The whole-body [^18^F]T-008 image series were reconstructed using standard filtered back-projection with standard corrections for random, scatter, and attenuation provided by the camera manufacturer. Prior to PET, each animal received a whole-body computed tomography scan that was used for attenuation and scatter correction and to assist in anatomical identification of organ of interest.

Reconstructed image data volumes were transferred to the PMOD in which volumes of interest were manually drawn on the following source organs: brain, kidneys, heart, urinary bladder, liver, spleen, vertebrae (bone marrow), lungs, intestine, testes (male), uterus (female), and gallbladder. Total activity (kBq) within each volume of interest was determined and the decay corrected and non-decay corrected time activity curves were generated and expressed as percent of the injected activity.

Radiation absorbed dose, effective dose equivalent (weighted average of the absorbed dose with radiation weighting factors from International Commission on Radiological Protection [ICRP] publication 26) and effective dose (weighting factors from ICRP publication 60) were estimated with OLINDA | EXM 1.1 software using the non-decay corrected time-activity data obtained for the identified source organs. The number of disintegrations per unit activity administered in each source organ (also known as residence times) for humans were computed from rhesus values by using conversion factors based on differences in organ and whole-body weights between the two species (Supplemental Table 2). OLINDA | EXM performs internal dose calculations based on Medical Internal Radiation Dose (MIRD) and Radiation Dose Assessment Resource methodology. ICRP publication 30 gastrointestinal tract kinetics were used with the assumption that activity entered the small intestine with no reabsorption. The adult male (73 kg) and female (57 kg) models were assumed for referent s-factors. All other MIRD assumptions with regard to the homogeneity of source organ distribution were employed.

# Chemical synthesis

**(3-Fluoroazetidin-1-yl)(1-(4-(4-fluorophenyl)pyrimidin-5-yl)piperidin-4-yl)methanone (T-008)**. A mixture of **6** (1.20 g, 3.98 mmol), 3-fluoroazetidine hydrochloride (0.533 g, 4.78 mmol), HATU (1.82 g, 4.78 mol), DIPEA (1.73 mL, 9.96 mmol) and DMF (10 mL) was stirred at room temperature (rt) for 18 h. The mixture was diluted with EtOAc and water, and then extracted with EtOAc. The organic layer was separated, washed with water and brine, dried over Na_2_SO_4_, and concentrated in vacuo. The residue was purified by column chromatography (NH silica gel, EtOAc/hexane) and recrystallized from EtOAc/heptane to give **T-008** (842 mg, 2.35 mmol, 59%) as white solids. ^1^H NMR (300 MHz, CDCl_3_) δ 1.61–1.94 (4H, m), 2.16–2.31 (1H, m), 2.59–2.73 (2H, m), 3.22–3.35 (2H, m), 4.01–4.50 (4H, m), 5.18–5.47 (1H, m), 7.12–7.22 (2H, m), 8.09–8.18 (2H, m), 8.41 (1H, s), 8.90 (1H, s). MS (ESI/APCI) m/z 359 [M+H]^+^. Anal. Calcd for C_19_H_20_F_2_N_4_O: C, 63.68; H, 5.62; N, 15.63. Found: C, 63.69; H, 5.68; N, 15.52.

**(1-(4-(3-Bromo-4-fluorophenyl)pyrimidin-5-yl)piperidin-4-yl)(3-fluoroazetidin-1-yl)methanone (1)**. A mixture of **12** (2.00 g, 5.26 mmol), 3-fluoroazetidine hydrochloride (0.704 g, 6.31 mmol), HATU (2.40 g, 6.31 mol), DIPEA (2.29 mL, 13.2 mmol) and DMF (20 mL) was stirred at rt for 2 h. The mixture was diluted with EtOAc and water, and then extracted with EtOAc. The organic layer was separated, washed with water and brine, dried over Na_2_SO_4_, and concentrated in vacuo. The residue was purified by column chromatography (NH silica gel, eluted with 0%–100% EtOAc in hexane) and recrystallized from EtOAc/heptane to give **1** (1.95 g, 4.46 mmol, 85%) as white solids. ^1^H NMR (300 MHz, CDCl_3_) δ 1.64–1.97 (4H, m), 2.19–2.33 (1H, m), 2.62–2.78 (2H, m), 3.21–3.34 (2H, m), 4.02–4.54 (4H, m), 5.17–5.49 (1H, m), 7.22 (1H, t, J = 8.4 Hz), 8.16 (1H, ddd, J = 8.6, 4.8, 2.2 Hz), 8.39 (1H, dd, J = 6.8, 2.1 Hz), 8.44 (1H, s), 8.91 (1H, s). MS (ESI/APCI) m/z 437.1 [M+H]^+^. Anal. Calcd for C_19_H_19_BrF_2_N_4_O:
C, 52.19; H, 4.38; N, 12.81. Found: C, 52.09; H, 4.24; N, 12.84.

**1-((1-(4-(4-Fluorophenyl)pyrimidin-5-yl)piperidin-4-yl)carbonyl)azetidin-3-yl4-methylbenzenesulfonate (2)**. A mixture of **7** (674 mg, 1.89 mmol), TsCl (541 mg, 2.84 mmol), Et_3_N (0.791 mL, 5.67 mmol), trimethylamine hydrochloride (36.1 mg, 0.380 mmol) and MeCN (15 mL) was stirred at rt for 30 min. The mixture was diluted with water and the resultant precipitates were collected, washed with water and EtOAc, and dried in vacuo. The crude solids were recrystallized from EtOAc/heptane to give **2** (305 mg,
0.597 mmol, 32%) as white solids.^1^H NMR (300 MHz, CDCl_3_) δ1.61–1.89 (4H, m), 2.11–2.24 (1H, m), 2.47 (3H, s), 2.57–2.70 (2H, m), 3.20–3.33 (2H, m), 3.92 (1H, dd, J = 11.6, 4.1 Hz), 4.16 (1H, dd, J = 11.0, 6.9 Hz), 4.26 (1H, dd, J = 10.1, 3.5 Hz), 4.37–4.46 (1H, m), 5.02–5.11 (1H, m), 7.11–7.20 (2H, m), 7.35–7.42 (2H, m), 7.76–7.82 (2H, m), 8.07–8.17 (2H, m), 8.40 (1H, s), 8.90 (1H, s). MS (ESI/APCI) m/z 511 [M+H]^+^. Anal. Calcd for C_26_H_27_FN_4_O_4_S: C, 61.16; H, 5.33; N, 10.97. Found: C, 60.95; H, 5.34;
N, 10.83.

**Ethyl 1-(2-(4-fluorophenyl)-2-oxoethyl)piperidine-4-carboxylate (4).** To a mixture of ethyl piperidine-4-carboxylate (8.02 g, 51.0 mmol)<autotext key="0F6A613C" name="[Reactants]" index="2" field="Reactants" type="field" length="51" /> and K_2_CO_3_ (9.61 g, 69.5 mmol)<autotext key="0F6A613D" name="[Reactants]" index="3" field="Reactants" type="field" length="26" /> in CH_3_CN (90 ml) was added dropwise 2-chloro-1-(4-fluorophenyl)ethanone **3** (8.00 g, 46.4 mmol)<autotext key="0F6A613F" name="[Reactants]" index="1" field="Reactants" type="field" length="56" /> in CH_3_CN (60 mL) at rt<autotext key="0F6A6140" name="[degree]" type="lookup" length="19" />. The mixture was stirred at the same temperature<autotext key="0F6A6141" name="[degree]" type="lookup" length="23" /> overnight<autotext key="0F6A6143" name="[for time]" type="lookup" length="9" />. The reaction mixture was concentrated in vacuo, diluted with water, and extracted with EtOAc. The extract was washed with brine, dried over Na_2_SO_4_, filtered, and concentrated in vacuo to give **4** (13.2 g, 45.0 mmol, 97%)<autotext key="0F9B2651" name="[Products]" index="1" field="Products" type="field" length="89" /> as a pale-yellow solid. This product was subjected to the next reaction without further purification. MS (ESI) *m/z* 294.1 [M+H]^+^.

**Ethyl 1-(4-(4-fluorophenyl)pyrimidin-5-yl)piperidine-4-carboxylate (5).** A mixture of **4** (13.2 g,
45.0 mmol)<autotext key="0F6C0F63" name="[Reactants]" index="1" field="Reactants" type="field" length="84" /> and DMF-DMA (82 ml, 585 mmol)<autotext key="0F6C0F65" name="[Reactants]" index="2" field="Reactants" type="field" length="28" /> was refluxed overnight<autotext key="0F6C0F69" name="[for time]" type="lookup" length="9" />. The mixture was concentrated in vacuo. The mixture was dissolved into n-BuOH (40 ml)<autotext key="0F6E9251" name="[Solvents]" index="1" field="Solvents" type="field" length="14" /> and DIPEA (40 mL)<autotext key="0F6E9252" name="[Solvents]" index="2" field="Solvents" type="field" length="13" />. Formamidine acetate (16.4 g, 157.50 mmol)<autotext key="0F6E9253" name="[Reactants]" index="3" field="Reactants" type="field" length="42" /> was added to the mixture and the mixture was stirred at 100°C<autotext key="0F9B265B" name="[degree]" type="lookup" length="9" /> overnight. The mixture was diluted with water and extracted with EtOAc. The extract was washed with water and brine, dried over MgSO_4_, filtered, and concentrated in vacuo. The residue was purified by column chromatography (silica gel, eluted with 5%<autotext key="0F6E9255" name="[xx%]" type="lookup" length="2" />–50%<autotext key="0F6E9256" name="[xx%]" type="lookup" length="3" /> EtOAc in hexane) to give **5** (9.93 g, 30.1 mmol, 67%)<autotext key="0F6E9257" name="[Products]" index="1" field="Products" type="field" length="94" /> as a brown solid including some impurities. This product was subjected to the next reaction without further purification. MS (ESI) *m/z* 330.1 [M+H]^+^.

**1-(4-(4-Fluorophenyl)pyrimidin-5-yl)piperidine-4-carboxylic acid (6).** To a mixture of **5** (9.93 g,
30.2 mmol)<autotext key="0F9CCC46" name="[Reactants]" index="1" field="Reactants" type="field" length="87" /> in THF (80 ml)<autotext key="0F9CCC48" name="[Solvents]" index="1" field="Solvents" type="field" length="16" /> and MeOH (20 ml)<autotext key="0F9CCC4B" name="[Solvents]" index="2" field="Solvents" type="field" length="12" /> was added 2 M NaOH (30.1 ml, 60.3 mmol)<autotext key="0F9CCC49" name="[Reactants]" index="2" field="Reactants" type="field" length="29" /> at rt. After being stirred at the same temperature<autotext key="0F9CCC4C" name="[degree]" type="lookup" length="23" /> for 1.5 h, the mixture was concentrated in vacuo, diluted with water, and neutralized with 2 M HCl. The resulting solid was collected and dried in vacuo to give **6** (8.28 g,
27.5 mmol, 91%)<autotext key="0F9CCC50" name="[Products]" index="1" field="Products" type="field" length="90" /> as pale-yellow solids. (3 steps, 59%). ^1^H NMR (300 MHz, DMSO-d_6_) δ 1.49–1.68 (2H, m), 1.75–1.88 (2H, m), 2.24–2.39 (1H, m), 2.61–2.75 (2H, m), 3.05–3.19 (2H, m), 7.30–7.41 (2H, m), 8.08–8.20 (2H, m), 8.54 (1H, s), 8.85 (1H, s), 12.26 (1H, s). MS (ESI) *m/z* 302.1 [M+H]^+^.

**(1-(4-(4-Fluorophenyl)pyrimidin-5-yl)piperidin-4-yl)(3-hydroxyazetidin-1-yl)methanone (7)**. A mixture of **6** (0.700 g, 2.32 mmol), azetidin-3-ol hydrochloride (0.331 g, 3.02 mmol), HATU (1.15 g,
3.02 mmol), DIPEA (1.21 mL, 6.97 mmol), and DMF (7 mL) was stirred at rt for 5 h. The mixture was diluted with EtOAc and water, and then extracted with EtOAc. The organic layer was separated, washed with water and brine, dried over Na_2_SO_4_, and concentrated in vacuo. The residue was purified by column chromatography (NH silica gel, EtOAc/hexane; MeOH/EtOAc) to give **7** (0.677 g, 1.90 mmol, 82%) as pale-yellow solids. ^1^H NMR (300 MHz, CDCl_3_) δ 1.62–1.94 (4H, m), 2.17–2.31 (1H, m), 2.40 (1H, d,
J = 5.7 Hz), 2.58–2.72 (2H, m), 3.20–3.34 (2H, m), 3.87 (1H, dd, J = 10.6, 4.1 Hz), 4.01 (1H, dd, J = 9.1, 4.2 Hz), 4.20–4.43 (2H, m), 4.64–4.77 (1H, m), 7.13–7.21 (2H, m), 8.09–8.18 (2H, m), 8.40 (1H, s), 8.90 (1H, s). MS (ESI/APCI) m/z 357 [M+H]^+^.

**1-(3-Bromo-4-fluorophenyl)-2-chloroethanone (9).** A mixture of 3'-bromo-4'-fluoroacetophenone **8**
(5.31 g, 24.5 mmol), *N*-chlorosuccinimide (3.27 g, 24.5 mmol), *p*-toluenesulfonic acid monohydrate
(0.465 g, 2.45 mmol) and MeCN (50 mL) was refluxed for 18 h. After being cooled to 0^o^C, the mixture was neutralized with aq. NaHCO_3_ and extracted with EtOAc. The organic layer was separated, washed with water and brine, dried over Na_2_SO_4_, and concentrated in vacuo. The residue was purified by column chromatography (silica gel, eluted with 0%–10% EtOAc in hexane) to give **9** (4.73 g, 18.8 mmol, 77%) as a colorless crystal. ^1^H NMR (300 MHz, CDCl_3_) δ 4.64 (2H, s), 7.21–7.28 (1H, m), 7.93 (1H, ddd, J = 8.6, 4.7, 2.2 Hz), 8.20 (1H, dd, J = 6.5, 2.2 Hz).

**Ethyl 1-(2-(3-bromo-4-fluorophenyl)-2-oxoethyl)piperidine-4-carboxylate (10).** To a mixture of ethyl isonipecotate (3.19 mL, 20.7 mmol)<autotext key="0F6A613C" name="[Reactants]" index="2" field="Reactants" type="field" length="51" /> and K_2_CO_3_ (3.90 g, 28.2 mmol)<autotext key="0F6A613D" name="[Reactants]" index="3" field="Reactants" type="field" length="26" /> in MeCN (50 mL) was added dropwise **9** (4.73 g, 18.8 mmol)<autotext key="0F6A613F" name="[Reactants]" index="1" field="Reactants" type="field" length="56" /> in MeCN (50 mL) at rt<autotext key="0F6A6140" name="[degree]" type="lookup" length="19" />. The mixture was stirred at the same temperature<autotext key="0F6A6141" name="[degree]" type="lookup" length="23" /> for 1.5 h<autotext key="0F6A6143" name="[for time]" type="lookup" length="9" />. The reaction mixture was concentrated in vacuo, diluted with water, and extracted with EtOAc. The extract was washed with brine, dried over Na_2_SO_4_, filtered, and concentrated in vacuo to give **10**<autotext key="0F9B2651" name="[Products]" index="1" field="Products" type="field" length="89" /> as a pale-yellow oil. This product was subjected to the next reaction without further purification. MS (ESI/APCI) *m/z* 372.1 [M+H]^+^.

**Ethyl 1-(4-(3-bromo-4-fluorophenyl)pyrimidin-5-yl)piperidine-4-carboxylate (11).** A mixture of **10** (7.00 g, 18.8 mmol)<autotext key="0F6C0F63" name="[Reactants]" index="1" field="Reactants" type="field" length="84" /> and DMF-DMA (34.3 mL, 244 mmol)<autotext key="0F6C0F65" name="[Reactants]" index="2" field="Reactants" type="field" length="28" /> was refluxed for 24 h<autotext key="0F6C0F69" name="[for time]" type="lookup" length="9" />. The mixture was concentrated in vacuo. The mixture was dissolved into n-BuOH (20 mL)<autotext key="0F6E9251" name="[Solvents]" index="1" field="Solvents" type="field" length="14" /> and DIPEA (20 mL)<autotext key="0F6E9252" name="[Solvents]" index="2" field="Solvents" type="field" length="13" />. Formamidine acetate (6.85 g, 65.8 mmol)<autotext key="0F6E9253" name="[Reactants]" index="3" field="Reactants" type="field" length="42" /> was added to the mixture and the mixture was stirred at 100°C<autotext key="0F9B265B" name="[degree]" type="lookup" length="9" /> for 24 h. The mixture was diluted with water and extracted with EtOAc. The extract was washed with water and brine, dried over Na_2_SO_4_, filtered, and concentrated in vacuo. The residue was purified by column chromatography (silica gel, eluted with 0%<autotext key="0F6E9255" name="[xx%]" type="lookup" length="2" />–40%<autotext key="0F6E9256" name="[xx%]" type="lookup" length="3" /> EtOAc in hexane) to give **11** (3.94 g,
9.65 mmol, 51%)<autotext key="0F6E9257" name="[Products]" index="1" field="Products" type="field" length="94" /> as a pale-yellow oil (2 steps, 51% from **9**). ^1^H NMR (300 MHz, CDCl_3_) δ 1.27 (3H, t,
J = 8.0 Hz), 1.71–1.88 (2H, m), 1.90–2.02 (2H, m), 2.41 (1H, tt, J = 11.0, 4.0 Hz), 2.67–2.80 (2H, m), 3.15–3.28 (2H, m), 4.16 (2H, q, J = 8.0 Hz), 7.22 (1H, t, J = 8.5 Hz), 8.13 (1H, ddd, J = 8.6, 4.8, 2.3 Hz), 8.41–8.47 (2H, m), 8.91 (1H, s). MS (ESI/APCI) *m/z* 408.1 [M+H]^+^.

**1-(4-(3-Bromo-4- fluorophenyl)pyrimidin-5-yl)piperidine- 4-carboxylic acid (12).** To a mixture of **11** (3.94 g, 9.65 mmol)<autotext key="0F9CCC46" name="[Reactants]" index="1" field="Reactants" type="field" length="87" /> in THF (28 mL)<autotext key="0F9CCC48" name="[Solvents]" index="1" field="Solvents" type="field" length="16" /> and MeOH (7 mL)<autotext key="0F9CCC4B" name="[Solvents]" index="2" field="Solvents" type="field" length="12" /> was added 2 M NaOH (9.65 mL, 19.3 mmol)<autotext key="0F9CCC49" name="[Reactants]" index="2" field="Reactants" type="field" length="29" /> at rt. After being stirred at the same temperature<autotext key="0F9CCC4C" name="[degree]" type="lookup" length="23" /> for 12 h, the mixture was concentrated in vacuo, diluted with water, and neutralized with 2 M HCl (9.65 mL). The resulting solid was collected and dried in vacuo to give **12** (3.44 g, 9.05 mmol, 94%)<autotext key="0F9CCC50" name="[Products]" index="1" field="Products" type="field" length="90" /> as off-white solids. ^1^H NMR (300 MHz, DMSO-d_6_) δ 1.50–1.67 (2H, m), 1.78–1.90 (2H, m), 2.24–2.40 (1H, m), 2.63–2.79 (2H, m), 3.04–3.22 (2H, m), 7.54 (1H, t, J = 8.7 Hz), 8.17 (1H, ddd, J = 8.7, 4.9, 2.1 Hz), 8.44 (1H, dd, J = 6.9, 2.1 Hz), 8.58 (1H, s), 8.87 (1H, s), 12.26 (1H, brs). MS (ESI/APCI) *m/z* 380.1 [M+H]^+^.
